# Music and social bonding: “self-other” merging and neurohormonal mechanisms

**DOI:** 10.3389/fpsyg.2014.01096

**Published:** 2014-09-30

**Authors:** Bronwyn Tarr, Jacques Launay, Robin I. M. Dunbar

**Affiliations:** Social and Evolutionary Neuroscience Research Group, Department of Experimental Psychology, University of OxfordOxford, UK

**Keywords:** music, rhythm, social bonding, endorphins, self-other merging, synchrony

## Abstract

It has been suggested that a key function of music during its development and spread amongst human populations was its capacity to create and strengthen social bonds amongst interacting group members. However, the mechanisms by which this occurs have not been fully discussed. In this paper we review evidence supporting two thus far independently investigated mechanisms for this social bonding effect: self-other merging as a consequence of inter-personal synchrony, and the release of endorphins during exertive rhythmic activities including musical interaction. In general, self-other merging has been experimentally investigated using dyads, which provide limited insight into large-scale musical activities. Given that music can provide an external rhythmic framework that facilitates synchrony, explanations of social bonding during group musical activities should include reference to endorphins, which are released during synchronized exertive movements. Endorphins (and the endogenous opioid system (EOS) in general) are involved in social bonding across primate species, and are associated with a number of human social behaviors (e.g., laughter, synchronized sports), as well as musical activities (e.g., singing and dancing). Furthermore, passively listening to music engages the EOS, so here we suggest that both self-other merging and the EOS are important in the social bonding effects of music. In order to investigate possible interactions between these two mechanisms, future experiments should recreate ecologically valid examples of musical activities.

## INTRODUCTION

Music-making, and movement to music, are activities central to ritual, courtship, identity, and human expression cross-culturally. Based on this ubiquity, it is argued that music has played an important role during our evolutionary history (Cross, 2001; Huron, 2001; McDermott, 2008; Dunbar, 2012b; although see, Pinker, 1997 for an alternative perspective).Whilst sexual selection and courtship are proposed as partial explanations for the widespread appreciation and aptitude for music (Brown, 2000; Merker, 2000; Miller, 2000 for a critique), there are other suggestions regarding its positive role for human societies. In this review we focus on the fact that in almost all cultures globally, and throughout history, music is a social activity (Nettl, 1983, 2000) that involves movement to rhythmic sound and plays a significant role both in creating social bonds (Roederer, 1984; McNeill, 1995; Freeman, 2000; Dunbar, 2004) and indicating coalition strength (Hagen and Bryant, 2003). This effect of musical activity on “social bonding” (the psychological experience of increased social closeness, reflected in prosocial behaviors) may be responsible for the widespread occurrence of musical activities and may have played an important role in the evolution of human sociality (Dunbar, 2012a,b).

While there has been much interest in the relationship between music and social bonding, there is as yet no consensus about the mechanisms by which this might occur. Many aspects of music-making which make people feel socially close are not specific to music-based activities, such as sharing attention with co-actors (e.g., Reddy, 2003), working toward similar goals (e.g., Tomasello et al., 2005), and experiencing a sense of positivity after successful co-engagement (e.g., Isen, 1970). An important feature that distinguishes musical activities from other social behavior is the importance of shared rhythms, and the externalization of predictable rhythms that allow synchronization to occur between two or more people (e.g., Bispham, 2006; Merker et al., 2009). Furthermore, people attribute movement and human agency to musical sound (e.g., Cross, 2001), which influences how synchronization occurs (Launay et al., 2013, 2014) as well as impacting upon affective experience (e.g., Fritz et al., 2013a). Here we focus on two proposed mechanisms of social bonding: self-other merging as a consequence of interpersonal synchrony, and the release of endorphins during synchronized exertive movements. We bring together evidence that both pathways from music-making to social bonding are relevant, highlight connections between the two, and suggest that both should be included in any account of how people form and maintain social bonds through music-making.

Firstly, performing movements simultaneously with someone else, (i.e., synchronizing), is believed to cause some blurring of self and other via neural pathways that code for both action and perception (Overy and Molnar-Szakacs, 2009). Secondly, it has been argued that group music-making leads to social bonding due to the release of neurohormones, specifically oxytocin (e.g., Freeman, 2000; Huron, 2001; Grape et al., 2003). The oxytocin account relies on its action as a social neurohormone in a range of mammals (e.g., Insel, 2010), and the suggestion that music-making (which involves sensory overload, physical activity, strong emotional arousal and social behavior) is particularly conducive to oxytocin release (e.g., Freeman, 2000). Whilst elevated oxytocin levels has been linked to increased trust (Kosfeld et al., 2005; Zak et al., 2005), eye contact (Guastella et al., 2008), face memory (Savaskan et al., 2008), generosity (Zak et al., 2007), empathy and the ability to infer the mental state of others (Domes et al., 2007), the causal link between music-related physical experience and oxytocin described by Freeman is tenuous. Here we review the evidence that the endogenous opioid system (EOS), and particularly endorphins, play a central role in the maintenance of non-sexual, non-kinship social bonds (Machin and Dunbar, 2011) that are characteristic of group musical activities. Given that endorphins are argued to mediate the pleasure experienced when listening to music (e.g., Huron, 2006; Koelsch, 2010) and recent evidence demonstrates that endorphins are released during synchronized and exertive activity (Cohen et al., 2010; Sullivan and Rickers, 2013; Sullivan et al., 2014), we argue that this particular peptide is an important candidate for the neurohormonal underpinnings of social bonding during group musical activities.

To begin, we will explore the evidence linking synchronization and social bonding, and subsequently the particular role of self-other merging, which may occur via shared neural pathways for action and perception. Following this we review evidence of the EOS’s role in social bonding, and discuss the case for this mechanism in musical activities. Finally we highlight the importance of using ecologically valid musical contexts in future investigation into the possible relationship between the two mechanisms that underpin the relationship between music and social bonding.

## SYNCHRONIZATION AND SOCIAL BONDING

Synchronization is often cited as an important mechanism by which social bonding can occur (Hove and Risen, 2009; Wiltermuth and Heath, 2009; Valdesolo and Desteno, 2011; Launay et al., 2013). This proposition builds in part on an identified relationship between mimicry (i.e., making a similar movement to another individual) and positive social behavior, such as self-reported rapport between two individuals (e.g., LaFrance and Broadbent, 1976; LaFrance, 1979). Mimicry improves rapport between people (Chartrand and Bargh, 1999; Lakin and Chartrand, 2003), which in turn influences the amount of mimicry that people perform (Van Baaren et al., 2004; Stel et al., 2010), thereby causing a positive feedback loop in which people can become increasingly socially close to one another through making similar movements, and more inclined to continue making similar movements once social closeness is established. Synchrony, like mimicry, involves simultaneous movements with another individual, with the additional element of rhythmically matched timing, which requires the prediction of movements of co-actors. Consequently, synchronization is likely to have similar or more pronounced effects on social bonding than mimicry.

People tend to spontaneously and unintentionally synchronize movements with one another, even to some extent when instructed not to do so (Issartel et al., 2007; Oullier et al., 2008; van Ulzen et al., 2008). Those with pro-social tendencies exhibit more spontaneous synchronization than those with pro-self tendencies (Lumsden et al., 2012), and the desirability of a partner can influence whether synchrony occurs (Miles et al., 2010, 2011), suggesting that this is a social behavior, rather than an automatic motor process. Perception of synchrony is also interpreted as a signal of rapport for both basic sounds (e.g., sound of people walking together: Miles et al., 2009; Lakens and Stel, 2011), and more complex musical stimuli (Hagen and Bryant, 2003).

More importantly, there is evidence that synchronization between people can influence their subsequent positive social feelings toward one another. This has been demonstrated in a number of experimental studies, involving participants tapping synchronously with an experimenter (Hove and Risen, 2009; Valdesolo and Desteno, 2011), walking in time with other people (Wiltermuth and Heath, 2009; Wiltermuth, 2012), dancing together (Reddish et al., 2013), and even when people have no visual access to one another but are synchronizing with the sounds of another person (Kokal et al., 2011; Launay et al., 2014).

The likely importance of social bonding via synchrony in *music*-based activities draws on the observation that beyond a tendency to synchronize with one another, humans have a culturally ubiquitous aptitude for entrainment to rhythmic beats (Clayton et al., 2005; Brown and Jordania, 2011), particularly those embedded in music (e.g., Demos et al., 2012). However, the source and context associated with those rhythms are paramount. For example, Kirschner and Tomasello (2009) demonstrated that children’s synchronization with a beat is improved in the presence of a person compared to when interacting solely with an isochronously beating drum. This suggests that from a young age, the awareness of agency related to perceived sound (and belief that the sound is produced by the intentional movements of another person) encourages synchronization with that sound, thereby likely influencing the social bonding effects of musical activities. Agent-driven sounds, and the associated perception of movement of another person, engage motor regions in the listener’s brain, potentially resulting in “self-other merging,” which has been argued to arise when individuals experience their movement simultaneously with another’s.

## SELF-OTHER MERGING AND SOCIAL BONDING

When moving at the same time as others we experience some co-activation of neural networks that relate to movement of self (as action), and other (as perception; e.g., Overy and Molnar-Szakacs, 2009). There is much recent research investigating the relationship between perception and action (Buccino et al., 2001; Fadiga et al., 2002; Rizzolatti, 2005; Caetano et al., 2007), which has identified “mirror neurons” in macaques (Gallese et al., 1996; Rizzolatti et al., 1996) that selectively respond to the macaque’s own movement and perception of the goal-directed movement of others. While there is no evidence for neurons with equivalent selectivity in humans (Hickok, 2009), this research led to much interest into how perception of goal-directed movement can engage regions of the brain related to making similar movements (Rizzolatti, 2005). Importantly it is now well recognized that perceiving the actions of another person can lead to activation of the same neural motor networks involved in making those actions oneself (e.g., Fadiga et al., 1995).

When our own actions match those of another’s, it is possible that the intrinsic and extrinsic engagement of neural action-perception networks make it difficult to distinguish between self and perceived other, thus creating at least a transient bond between the two (Decety and Sommerville, 2003; Sebanz et al., 2006; Sommerville and Decety, 2006; Knoblich and Sebanz, 2008; Marsh et al., 2009; Overy and Molnar-Szakacs, 2009). A well replicated experimental example of this is the rubber hand illusion (Botvinick and Cohen, 1998). In this paradigm, a participant’s arm is hidden from sight, and a replacement rubber arm is visible where their own arm is expected. While they view the rubber hand being touched with a paintbrush, their own (hidden) hand is simultaneously touched with a paintbrush, with synchronized strokes. This matching of visual and tactile input leads to an increased subjective sense that the rubber hand is part of the participant’s body. The effect disappears when the two inputs are not synchronized. This provides evidence that self-other blurring is possible even with an inanimate object, and some aspects of this are likely to apply to human–human synchronized interaction. Indeed, behavioral synchrony has also been demonstrated to induce common neural signatures between interacting agents (Oullier et al., 2005; Tognoli et al., 2007; Lindenberger et al., 2009; Dumas et al., 2010). However, evidence for common neural signatures during synchronization should be interpreted with caution, as it can only indicate that similar cortical networks are involved in making the same movements for different people.

Researchers who argue that self-other merging is an important part of the bonding effects of synchronization primarily draw support from dyadic experiments in which participants’ actions are perceived to occur at the same time as one another. Theoretically, dyads are capable of achieving synchrony with relative ease simply because there is only one other person to keep track of. As such, synchrony is reasonably attainable, and associated self-other merging (and bonding) effects are likely to be achieved fairly easily.

Musical activities, on the other hand, are not limited to one-on-one interactions, and have historically involved groups (Nettl, 1983, 2000). With large numbers of people, it is difficult to simultaneously observe the movements of all the other participants, making self-other merging a less likely prospect. Rhythm provides an external, predictable scaffolding that can facilitate synchrony with both the music, and by extension, aids synchrony between individuals engaging in the same musical experience. A recent experiment involved people rocking on rocking chairs with one another, while music played in the room or did not (Demos et al., 2012). While self-reported rapport between co-actors correlated with synchronization achieved with the *music*, rapport did not correlate with synchronization that occurred between co-actors. This implies that externalizing the target of synchrony (e.g., to music) allows bonding with other people present, in the absence of explicit synchrony between those people. This finding has important implications given that group musical activities often involve non-identical movements between people (making self-other merging an unlikely prospect).

Given that the self-other merging account of social bonding relies on simultaneous, similar movements, it is likely that this mechanism does not provide a complete account for the bonding that arises in large group situations. Additional mechanisms need to be considered, in particular mechanisms that underpin the social bonding associated with musical activities. One likely mechanism involves the EOS, and particularly endorphins, which are released through synchronous and exertive activities, and during passive engagement with music, and play a central role in social bonding among primate species (e.g., Keverne et al., 1989).

## ENDORPHINS AND SOCIAL BONDING

Investigation into the neuropeptide underpinnings of social bonding have implicated neurohormonal cascades involving oxytocin and vasopressin (e.g., Carter, 1998), dopamine and serotonin (e.g., Depue and Morrone-Strupinsky, 2005), and endorphins released by the EOS (Curley and Keverne, 2005; Dunbar, 2010). Recently, oxytocin has been promoted as *the* social neurohormone (Bartz et al., 2011; Meyer-Lindenberg et al., 2011), largely due to evidence from pair-bonding and mother-infant bonding (e.g., Atzil et al., 2011; Feldman, 2012). However, despite apparent interactions between opioids (specifically endorphins) in the bonding activity of oxytocin (e.g., Depue and Morrone-Strupinsky, 2005), and evidence of the EOS’s role in primate pair-bonding (Ragen et al., 2013), maternal care (Martel et al., 1993), as well as empirical evidence that increased opioid levels are associated with social grooming and affiliative behaviors in non-sexual, non-kin related conspecifics (Keverne et al., 1989; Schino and Troisi, 1992; Martel et al., 1995), the role of the EOS in social bonding remains relatively underexplored, possibly due to the difficulties in measuring endorphin titres directly (Dearman and Francis, 1983).

The EOS consists of opioid receptors and associated ligands distributed throughout the central nervous system and peripheral tissues, such as the nucleus accumbens (Fields, 2007; Trigo et al., 2010). The EOS is central in opioid-mediated reward (Koob, 1992; Olmstead and Franklin, 1997; Comings et al., 1999), social motivation (Chelnokova et al., 2014), and pleasure and pain perception (Janal et al., 1984; Leknes and Tracey, 2008). Elevated opioid levels are correlated with feelings of euphoria (Boecker et al., 2008), and Koepp et al. (2009) report activation of general opioid receptors in the hippocampus and amygdala in response to positive affect. Deactivation of certain opioid receptor sites has been associated with negative affect (Zubieta et al., 2003).

The possible role of the EOS in social bonding is formalized in the brain opioid theory of social attachment (BOTSA). BOTSA is based on evidence of behavioral and emotional similarities between those in intense relationships, and those addicted to narcotics (Insel, 2003). Furthermore, endogenous opioids, particularly endorphins, are related to social bonding in many non-human animals such as rhesus macaques (Schino and Troisi, 1992; Graves et al., 2002), other monkeys (Keverne et al., 1989; Martel et al., 1995; Ragen et al., 2013), voles (Resendez et al., 2013), puppies, rats and chicks (Panksepp et al., 1980), and mammals generally (Broad et al., 2006). Given the role of endorphins in bonding in other species, it is plausible that the EOS may also underpin human social bonds (Matthes et al., 1996; Moles et al., 2004; Depue and Morrone-Strupinsky, 2005; Dunbar, 2010).

Opioids are released in response to low levels of muscular and psychological stress (Howlett et al., 1984), for example during exercise (Harbach et al., 2000). Positron emission tomography (PET) scans have confirmed the euphoric state that follows exercise (termed “runner’s high”) is due to endogenous opioids (Boecker et al., 2008). Further to the effect on mood, opioids have an analgesic effect (Van Ree et al., 2000), and much evidence suggests that endorphins are central in the pain management system (D’Amato and Pavone, 1993; Benedetti, 1996; Zubieta et al., 2001; Fields, 2007; Bodnar, 2008; Dishman and O’Connor, 2009; Mueller et al., 2010). Given that direct measures of endogenous opioids are costly and invasive (Dearman and Francis, 1983), pain threshold is a commonly used proxy measure of endorphin release, and this has been operationalised using the length of time holding a hand in ice water (Dunbar et al., 2012a,b), a ski exercise (maintaining a squat position with legs at right angles: Dunbar et al., 2012a), an electrocutaneous simulator (Jamner and Leigh, 1999), pressure produced using a blood pressure cuff (Cogan et al., 1987; Cohen et al., 2010; Dunbar et al., 2012a,b), and the amount of pain medication requested by patients (Zillmann et al., 1993).

According to pain threshold assays, various exertive human social bonding activities, such as laughter (Dezecache and Dunbar, 2012; Dunbar et al., 2012a), group synchronized sport (Cohen et al., 2010; Sullivan and Rickers, 2013), and singing and dance (Dunbar et al., 2012b), trigger endorphin release. Specifically, *synchronized* exertive activity (such as rowing) elevates pain thresholds significantly more than non-synchronized exertion (Sullivan and Rickers, 2013; Sullivan et al., 2014), suggesting that rhythmic, music-based activities may similarly facilitate endorphin release.

## ENDORPHINS AND MUSIC

Based on the association between exertion and endorphin release, a number of studies have investigated the effect of active engagement in musical activities (i.e., involving overt movement) and the EOS (see **Table 1**). For example, sufficiently vigorous singing, dancing, and drumming trigger a significantly larger increase in pain threshold and positive affect compared to listening to music and engaging in low energy musical activities (Dunbar et al., 2012b). In a recent set of studies, exercise machines were linked to musical output software such that individuals “created” music as they exerted themselves (Fritz et al., 2013a,b). These experiments demonstrated that when movement (during group exercise) results in musical feedback, participants perceived exertion to be lower (Fritz et al., 2013b), reported enhanced mood, and felt a greater desire to exert themselves further (Fritz et al., 2013a), in comparison to when they were exercising whilst listening (passively) to independently provided music. As such, perception of agency in a musical setting is associated with greater endorphin activation and may therefore lead to greater effects in terms of mood and ability to withstand strenuous exercise.

**Table 1:** Summary of studies providing evidence for the role of EOS in music-related activities.

	Passive listening	Active engagement
Pain threshold, pain management	Post-operative pain: Koch et al. (1998), Allen et al. (2001), Good et al. (2001), Lepage et al. (2001), Nilsson et al. (2001, 2003), Nilsson (2008), Bernatzky et al. (2011) for a review see Cepeda et al. (2006)	Singing, drumming, dance: Dunbar et al. (2012b)
Brain activation regions	EOS, pleasure, and reward circuits: Blood and Zatorre (2001), Stefano et al. (2004), Menon and Levitin (2005) Nucleus accumbens and pleasure states: Koelsch (2014)	
Emotions and mood	Techno-music: Gerra et al. (1998) Emotional effects of music: Koelsch (2010) Positive affect: Huron (2006)	Increased positive affect: Dunbar et al. (2012b) Enhanced mood: Fritz et al. (2013a)
Health	Lower blood pressure and relaxation: Chiu and Kumar (2003), Stefano et al. (2004) Anxiolytic music: McKinney et al. (1997b)	
Other	Musical “thrills”: Goldstein (1980), Panksepp (1995), Menon and Levitin (2005)	Perception of exertion and desire to exert oneself: Fritz et al. (2013b)

However, activation of the EOS through music is not limited exclusively to situations involving exertion (see **Table 1**). Listening to music reportedly helps to manage pre-operative hypertension and psychological stress (Allen et al., 2001), reduces sedative requirements during spinal anesthesia (Lepage et al., 2001) and other surgical procedures (Koch et al., 1998), decreases perception of pain (Good et al., 2001; Nilsson et al., 2003) thereby diminishing the need for opioid agonists following operative care (Cepeda et al., 2006; Bernatzky et al., 2011), and improves post-operative recovery (Nilsson et al., 2001). Many of the experiments in this area directly attribute these results to the EOS, and given the strong role of opioid receptor activation in analgesia (Leknes and Tracey, 2008), the body of work linking music and pain may generally be considered convincing evidence of the role of opioid activation.

Positron emission tomography (PET) and functional magnetic resonance imaging (fMRI) research also provide evidence that passive listening to music activates the EOS and brain areas associated with pleasure and reward (Blood and Zatorre, 2001; Stefano et al., 2004). For example, recent evidence that music listening is associated with activation in areas such as the nucleus accumbens (Brown et al., 2004; Menon and Levitin, 2005; Koelsch, 2014), the high number of opioid receptors in this region (Fields, 2007; Trigo et al., 2010) and the role of opioids in mood and pleasure states (Berridge and Kringelbach, 2008) provide support for the theory that the EOS is involved in music listening.

The importance of the EOS in regulating affective experiences in response to music (Zubieta et al., 2003) is further supported by evidence linking music induced “thrills” to endorphin activation (Goldstein, 1980), and the EOS’s association with reward circuits (Menon and Levitin, 2005). In addition, the sense of elation that arises when engaging in musical activities has been attributed to endorphin release (Chiu and Kumar, 2003; Huron, 2006; Dunbar, 2009). Calming music is thought to act via the EOS by buffering the effect of stressful events (see McKinney et al., 1997b for a review), and relaxation following music listening is also linked to the EOS (Stefano et al., 2004). Gerra et al. (1998) report that listening to techno-music significantly changes emotional states (and increases beta-endorphin levels), due to its strong rhythmic beat and engagement of motor regions of the brain. Activation of the EOS, and its role in various affective, calming and analgesic effects, is therefore evident in cases of passive music listening, although a systematic investigation of this effect is still lacking.

It is important to note that there is also some evidence indicating that neurohormones other than endorphins are involved during music-based activities (e.g., Grape et al., 2003; Bachner-Melman et al., 2005; Chanda and Levitin, 2013). In a recent review, Chanda and Levitin (2013) highlight evidence suggesting that stress and arousal effects associated with music-based activities can be linked to cortisol, corticotrophin-releasing hormone and adrenocorticotropic hormone (e.g., McKinney et al., 1997a; Gerra et al., 1998). Various immunity benefits of music have been attributed to, *inter alia*, cortisol (e.g., Beck et al., 2000; Kuhn, 2002), cytokinin (e.g., Stefano et al., 2004), and growth hormones (e.g., Gerra et al., 1998). Finally, dopamine is key in reward and motivation circuits during musical activities (e.g., Salimpoor et al., 2011), which are likely to interact synergistically with the EOS in mediating the pleasure states associated with music (Chanda and Levitin, 2013). While we argue for further investigation of the EOS as a potential mediator of the positive social effects of musical engagement, it may be difficult to separate out the role of this hormone from other neurochemicals involved in these experiences.

As indicated by the evidence reviewed above, the way that we experience music, whether during passive listening or active engagement, appears to involve the EOS, and endorphins specifically. In the following section we discuss how both self-other merging and the EOS mechanisms might underpin our musical experiences.

## FROM MUSIC TO SOCIAL BONDING

**FIGURE 1:**
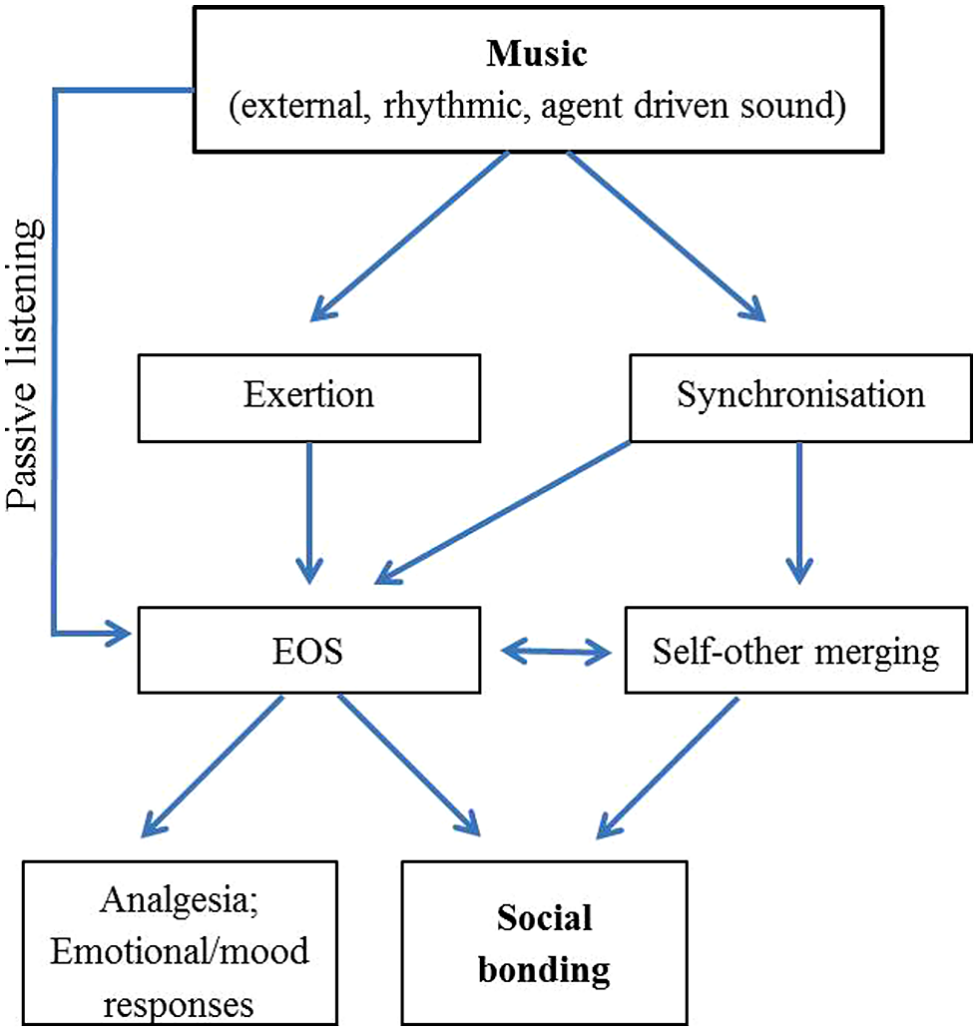
**Schematic diagram illustrating possible direction of causality, and relationship between, mechanisms behind the social bonding effects of music**.

Both self-other merging and the EOS help explain the subjective experience of social bonding that can arise during musical activities, as illustrated by **Figure 1**. However, as these two mechanisms have thus far been independently investigated, the interplay between them remains unclear.

As mentioned previously, dyads are capable of achieving synchrony with relative ease (even without music), while in larger groups, synchrony is facilitated via rhythmic scaffolding. Additionally, as music encourages movement (Janata and Grafton, 2003; Madison, 2006; Madison et al., 2011; Janata et al., 2012) by engaging motor regions of the brain (Levitin and Menon, 2003), we might expect engagement with musical sounds to be more exertive than with non-musical sounds. The combination of larger movements and the externalization of the target of synchrony likely facilitates synchronization.

Exertive movements cause affiliative sentiments and behaviors (e.g., Mueller et al., 2003), have effects on mood and emotion (e.g., Karageorghis and Terry, 2009), and, in combination with synchrony, can elevate pain thresholds (e.g., Cohen et al., 2010). These phenomena are all strongly associated with the EOS (e.g., Dishman and O’Connor, 2009). Accordingly, we propose that self-other matching and activation of the EOS are interconnected in explaining the bonding effects that arise during active engagement in group music-based activities, with a possibility that the EOS underpins the psychological experience of self-other merging.

In terms of passive listening to music, the literature reviewed here suggests that the EOS is likely to play a role also in the absence of explicit movement and self-other merging during synchrony. Dynamic attending theory (e.g., Jones and Boltz, 1989) suggests that through monitoring of events occurring with predictable temporal patterns we can become entrained to those events. This rhythmic predictability has been suggested to play a key role in the pleasure experienced when listening to music, which may be mediated by the release of endorphins (Huron, 2006; Margulis, 2013). The effect of tempo on arousal (Husain et al., 2002) and the strong ability for music to alter mood (Thayer et al., 1994) and motivational states (Frijda and Zeelenberg, 2001) are both congruous with evidence that EOS activation occurs in the brain when listening and entraining to music (Blood and Zatorre, 2001; Stefano et al., 2004; Menon and Levitin, 2005). Sievers et al. (2013) demonstrate that movement and music are processed cross-modally, as are the emotions expressed through movement and music. Elements of music significantly affect various dimensions of imagery relating to motion (Eitan and Granot, 2006), and listening to music may itself induce thoughts about movement, whether conscious or subconscious (Chen et al., 2008; Levitin and Tirovolas, 2009; Clarke, 2011). Through activation of motor regions of the brain during music listening (Levitin and Menon, 2003), passive engagement with music likely triggers the same neural pathways involved in active engagement (i.e. movement) to music, including pathways implicating the EOS. This activity in motor regions of the brain during music perception is likely to underlie the self-reported experience of “embodied movement” even when listening and not moving to music (e.g., Peters, 2010).

## CONCLUSION

While most accounts of the relationship between music and social bonding have focused separately on self-other merging via synchrony or neurohormonal mechanisms, here we suggest that associations between the two need to be considered, especially when assessing large-scale musical activities. Future work should be directed toward ecologically valid musical experiences involving groups of people interacting with one another rather than dyadic interaction, exertive movements rather than small movements, and movements that are temporally co-ordinated rather than synchronized *per se*. Using these forms of musical activity it will become possible to explore the relative importance of self-other matching and EOS in music-based activities (including passive listening). Given that humans have significantly larger and more complex social networks than our primate cousins, research in this field will elucidate the means by which our species has the capacity to bond with large groups of conspecifics at the same time. It is likely that some combination of endorphin release and self-other merging lead to the social bonding effects of music, although the relationship between the two mechanisms remains to be sufficiently explored.

## Conflict of Interest Statement

The authors declare that the research was conducted in the absence of any commercial or financial relationships that could be construed as a potential conflict of interest.
